# Exogenous Hydrogen Sulfide Ameliorates Diabetic Myocardial Fibrosis by Inhibiting Cell Aging Through SIRT6/AMPK Autophagy

**DOI:** 10.3389/fphar.2020.01150

**Published:** 2020-07-31

**Authors:** Yaling Li, Maojun Liu, Xiong Song, Xia Zheng, Jiali Yi, Da Liu, Sen Wang, Chun Chu, Jun Yang

**Affiliations:** ^1^ Department of Cardiology, The First Affiliated Hospital of University of South China, Hengyang, China; ^2^ Department of Pharmacy, The Second Affiliated Hospital of University of South China, Hengyang, China

**Keywords:** hydrogen sulfide, myocardial fibrosis, cell aging, Sirt6/AMPK pathway, autophagy

## Abstract

Stress aging of myocardial cells participates in the mechanism of myocardial fibrosis (MF). Previous studies have shown that hydrogen sulfide (H_2_S) can improve MF, however the specific internal mechanism remains still unclear. Therefore, this study aims to explore whether H_2_S can improve myocardial cell aging induced by high glucose and myocardial fibrosis in diabetic rats by activating autophagy through SIRT6/AMPK. We observed that HG (high glucose, 33 mM) induced down-regulation of endogenous H_2_S-producing enzyme CSE protein expression, increased cell senescence, down-regulation of autophagy-related proteins Beclin1, Atg5, Atg12, Atg16L1, and inhibition of SIRT6/AMPK signaling pathway in H9c2 cardiomyocytes. H_2_S (NaHS: 400 μM) could up-regulate CSE protein expression, inhibit cell senescence, activate autophagy and SIRT6/AMPK signaling pathway. On the contrary, no above phenomena was achieved upon addition of CSE inhibitor PAG (dl-propargylglycine: mmol/L). In order to further elucidate the relationship between H_2_S and SIRT6/AMPK signaling pathway, dorsomorphin dihydrochloride (Dor), an inhibitor of AMPK signaling pathway, was added to observe the reversal of H_2_S’s inhibitory effect on myocardial cell aging. At the same, streptozotocin (STZ; 40 mg/kg) was injected intraperitoneally to build an animal model of diabetic SD rats. The results showed that myocardial collagen fibers were significantly deposited, myocardial tissue senescent cells were significantly increased and the expression of CSE protein was down-regulated, while SIRT6/AMPK signaling pathway and cell autophagy were significantly inhibited. H_2_S-treated (NaHS; 56 μmol/kg) could significantly reverse the above phenomenon. In conclusion, these findings suggest that exogenous H_2_S can inhibit myocardial cell senescence and improve diabetic myocardial fibrosis by activating CSE and autophagy through SIRT6/AMPK signaling pathway.

## Introduction

Diabetes mellitus (DM) is one of the most common metabolic diseases in the world. Current epidemiological data show that type 2 diabetes alone affects about 8% of adults in the world, and the incidence rate increases with age (Koopman et al., 2005). Diabetes mellitus can lead to distinct poor clinical prognosis of patients, which is closely associated with cardiovascular complications. Long-term high glucose can cause obvious myocardial damage in patients, even resulting in a common chronic cardiac complication-diabetic myocardial fibrosis (DCM).The main pathological changes of diabetic cardiomyopathy include myocardial fibrosis, which leads to cardiac dysfunction, eventually progresses to heart failure or even sudden death (Falcao-Pires and Leite-Moreira, 2012; Zheng et al., 2019). As an aging-related disease, some studies have found that myocardial damage is related to stress premature aging (Glance et al., 2014), some studies also confirmed that ROS production can be increased remarkably by oxidative stress, promoting premature senescence of myocardial cells after the exposure of myocardial cells to high glucose (Rota et al., 2006). At the same time, Annoni et al. also found that the expression of myocardial type I and type III collagen in aging rats was significantly increased (Annoni et al., 1998). Kwak HB team also observed through *in vivo* and *in vitro* experiments that the production of ROS in aging animals’ hearts was significantly increased, while overexpression of superoxide dismutase could slow down cardiac aging-related fibrosis, which further confirmed that cell aging was closely related to myocardial fibrosis (Kwak et al., 2015). However, myocardial cell aging presumably becomes a new target for interfering with diabetic myocardial fibrosis and treating DCM.

Sirtuin6 (SIRT6) as a member of NAD^+^ dependent deacetylase family, which has been proved to play an important role in intracellular homeostasis, DNA damage repair, and even cell senescence (Kim et al., 2018). Previous studies have shown that SIRT6 can improve diabetic retinopathy by delaying cell senescence, meanwhile the down-regulation of SIRT6 expression also participates in the pathogenesis of DCM (Kwak et al., 2015; Kanwal et al., 2019). Adenylate activated protein kinase (AMPK) is a key signal molecule that can regulate cell energy metabolism. It is a heterotrimer complex composed of catalytic subunits (1, 2, 3) and regulatory subunits (1, 2, 3). AMPK can be activated by SIRT6 through increasing the ratio of AMP/ATP, thus promoting AMPK phosphorylation and regulating fat metabolism (Elhanati et al., 2013). In addition, AMPK-autophagy pathway is also closely related to cell senescence (Wang et al., 2018). However, it is not clear whether SIRT6/AMPK participates in the aging mechanism of myocardial cells stimulated by high glucose through regulating autophagy, which remains to be further studied.

Endogenous hydrogen sulfide (H_2_S) is the third endogenous gas signal molecule discovered in recent years. It is mainly catalyzed by endogenous enzymes including cystathionine-lyase (CSE), cystathionine-synthase (CBS) and 3-mercaptopyruvate sulfur transferase with cysteine aminotransferase (3-MST/CAT). It has been proved that the properties of anti-apoptosis, anti-inflammation, anti-oxidation, autophagy regulation, and anti-ER stress were achieved. Recently, it has also been found to inhibit endothelial cell aging and vascular aging (Arumugam and Kennedy, 2018); however, the specific mechanism is still unclear. In this study, we investigated the SD rat model of diabetes induced by Streptozotocin (STZ; 40 mg/kg) and H9c2 cardiomyocytes cultured in high glucose. To explore the mechanism by which exogenous H_2_S inhibits cell aging and improves myocardial fibrosis, and may be related to the SIRT6/AMPK signaling pathway.

## Materials and Methods

### Reagents

The H9c2 cell line was obtained from ATCC (Manassas, VA,USA), sodium hydrogen sulfide (NaHS) was purchased from Sigma-Aldrich (St. Louis, MO, USA). Dorsomorphin dihydrochloride (HY-13418) was obtained from MCE. Glucose, streptozotocin (STZ), dl-propargylglycine (PAG) were obtained from Sigma-Aldrich (St. Louis, MO, USA). Dulbecco’s modified Eagle’s medium (DMEM 5.5 mM D-glucose), penicillin, streptomycin and fetal bovine serum (FBS) were from Hyclone (Grand Island, NY), antibodies against SIRT6, AMPK, p-AMPK, LC3A/B, BECLIN1, ATG5, ATG16L1, P53, P21, P16 were from Boster Biological Technology (Ltd. Wuhan, China. Bicin choninic Acid (BCA) Protein Assay kit, Enhanced Chemiluminescence Reagent kit, SDS-PAGE Gel Preparation kit and phenyl methyl sulfonyl fluoride (PMSF) were purchased from Beyotime Institute of Biotechnology(Shanghai, China), SPiDER-βGal was purchased from dojindo (Japan).

### Cell Culture and Treatment

The H9c2 cell line was obtained from ATCC (Manassas, VA,USA), The cells were cultured in DMEM medium with 5.5 mM D-glucose, supplemented with 10% calf serum(from Gibco, Grand Island, NY,USA) and in an atmosphere at 37°C with 5% CO_2_.When the cells grew to 1 × 10^6^, cardiomyocytes were inoculated into a six-well plate, and high glucose (33 mM) was added to establish a high glucose intervention culture cell model (Guo et al., 2018). In the H_2_S-treated group, exogenous H_2_S donor NaHS (400 µM) was pretreated for 0.5 h in order to confirm whether H_2_S activates autophagy by activating SIRT6/AMPK signaling pathway (Xu et al., 2015), AMPK inhibitor (Dorsomorphin dihydrochloride 10 µM) was added to Dor group after 0.5 h pretreatment with NaHS (400 µM), Sirt6 inhibitor OSS_128167 (100 μM) was added to OSS group after 0.5 h pretreatment with NaHS (400 µM), PAG (2 m mol/L) was added to PAG group after 0.5 h pretreatment with NaHS (400 µM), and then cardiomyocytes of all groups were treated with HG (33 m M) at 37° C for 24 h except the control group. For HG treatments, cells were inserted with DMEM containing 33 mM glucosse.

### Senescence Cell Detection

The 6-well plate cells were incubated with different treatments and washed three times with phosphate buffered saline (PBS), immobilized at room temperature with 4% formaldehyde (EMD Millipore, Billerica, MA, USA) for 10 min, washed three times with PBS, cultured cells were incubated at 37°C with SPiDER-Gal (Dojin) for 30 min, and finally the cells were observed under a microscope (Japan OLYMPUS Corporation). (Doura et al., 2016)

Rat heart tissue specimen sections (6 µm) were likewise taken, fixed at room temperature with 4% formaldehyde (EMD Millipore, Billerica, MA, USA) for 10 min, washed three times with PBS, incubated overnight in SA-Gal staining solution, and aging changes were observed using a microscope.

### Immunofluorescence Detection

H9c2 cardiomyocytes with 6-well plates were incubated with different treatments, then the medium was removed and washed with PBS. Immobilization with 4% formaldehyde solution for 10 min at room temperature, washing with PBS for three times, then incubation in a blocking solution (BSA) for 15 min, then overnight with the primary antibody diluted in the blocking solution, washing with PBS for 3 times the next day, and incubation with the second antibody in the blocking solution for 1 h and then observation under a microscope (Japan OLYMPUS Corporation).

### Animals and Treatment

Forty adult male SD rats, weighing (240 ± 20 g), from the Animal Laboratory of South China University, were given food and water freely under the conditions of 12 h day and night, temperature at 23°C and humidity at 60°C, according to the “Regulations on the Administration of Experimental Animals” issued by the State Science and Technology Commission.

Forty male SD rats were randomly divided into the following four groups: control group, STZ group (diabetic rat group), STZ+H_2_S group (H_2_S intervention group) and H_2_S group. STZ group and STZ+H_2_S group were fed with high fat diet for 4 weeks (Chao et al., 2018; Zhuo et al., 2018; Wang et al., 2019), and then injected 40 mg/kg STZ intraperitoneally for 12 h after fasting and drinking. T2DM rats were established. The rats in the control group were injected intraperitoneally with the same amount of normal saline once after four weeks of standard feeding. Blood was drawn from tail vein of rats and fasting blood glucose was detected by blood glucose monitor. If the plasma glucose level of rats was higher than 16.7 mmol/L 3 days after STZ injection, it was considered that the diabetic rat model was successfully established. In addition, we administered a 50% glucose solution to rats for oral glucose tolerance test by gavage. And through the fasting insulin level and blood glucose meter to detect the corresponding fasting blood glucose value to calculate the insulin resistance index to verify the success of DM modeling. Rats in STZ+H_2_S group and H_2_S group were given intraperitoneal injection of H_2_S donor NaHS(NaHS,56 μmol/kg/d), respectively, while rats in control group and STZ group were given intraperitoneal injection of the same amount of normal saline every day for 4 weeks.

### Echocardiographic Analysis

After 4 weeks, all animals were anesthetized by intraperitoneal injection of chloral hydrate (3 ml/kg), and the left ventricular structure and function were examined by transthoracic echocardiography. Left ventricular end diastolic diameter (LVEDD), left ventricular end systolic diameter (LVESD), left ventricular ejection fraction (LVEF) and left ventricular short axis shortening rate (LVFS) were measured to evaluate the changes of cardiac structure and function in rats.

### Glucose Tolerance Test

The rats were administered a 50% glucose solution by gavage for the oral glucose tolerance test. Tail vein samples were collected at 30, 60, and 120 min after administration.

### Insulin Resistance

Through the fasting insulin level and blood glucose meter to detect the corresponding fasting blood glucose value to calculate the insulin resistance (HOMA-IR).

### Masson Staining

All animals were anesthetized by intraperitoneal injection of chloral hydrate (3 ml/kg), then killed by cervical spine beheading and collected heart specimens, fixed with 4% paraformaldehyde saline and embedded in paraffin. Masson staining were carried out according to the instructions of the reagent manufacturer Rinse with tap water first, then dehydrate regularly with gradient alcohol, remove with xylene, and embed with paraffin. Subsequently, the sections are regularly dewaxed and hydrated, and finally stained with hematoxylin. Sections were placed under a microscope for observation.

### Van Gieson (VG) Staining

Routine dewaxing of slices. Stain with Weigert hematoxylin for about 10 to 20 min, wash back to blue for 5 to 10 min. Stain with VG staining solution reagent for 0.5 to 2 min. Discard the dye solution and directly differentiate with 95% alcohol. Anhydrous ethanol is dehydrated, xylene is transparent and sealed. Sections were placed under a microscope for observation.

### RT-qPCR Analysis

Total RNA was extracted from myocardial tissue of rats in each group with Trizol reagent (Invitrogen, Califoia, USA), and then the concentration of the extracted RNA was determined by ultraviolet spectrophotometer (Agilent Technologies, CA, USA). The integrity of RNA was analyzed by gel imaging system (Bio-Rad Laboratories, Inc., Hercules, CA, USA). Reverse transcription polymerase chain reaction kit (MBI Fermentas, the Republic of liqua) was used to reverse transcribe cDNA with total tissue mRNA as template and RT primers (Gen Script USA Inc., Nanjing, China). The expression level was detected by real-time fluorescence quantitative PCR with Taqman mi-RNA assay probe (Applied Biosystems, Shanghai, China) and standardized with endogenous U6 microRNA. The cDNA obtained was subjected to real-time PCR under the following circumstances: 50°C for 2 min, 95°C for 10 min, 95°C for 5 s, and 60°C for 30 s.

### Electron Microscopy

The myocardial tissue of rats in each group was cut into 50- to 100-nm-thick sections and fixed with 2.5% glutaraldehyde (China National Medicine Group Chemical Reagent Co., Ltd.) before operation, then fixed with 1% osmium tetroxide(Absin Bioscience Inc. Shanghai China), rinsed with Phosphoric acid rinse solution (Beyotime Institute of Biotechnology, Shanghai, China),stained with 3% uranium dioxide acetate and lead nitrate, and observed the ultrastructure of the tissue under TEM.

### Western Blot Analysis

Myocardial tissue or cultured cell proteins were extracted with cell lysis buffer containing protease inhibitor (RIPA: PMSF=9: 1), and then the content of extracted proteins was detected by BCA protein quantitative detection kit. The denatured protein was separated by SDS-PAGE electrophoresis and transferred to poly vinylidene fluoride (PVDF) membrane (Billerica, USA). At room temperature, it was blocked with TBST solution containing 5% milk, then incubated overnight with the primary antibody of the detected protein. After washing, it was incubated with the secondary antibody of anti-rabbit or anti-mouse IgG conjugated with horseradish peroxidase. ECL development photography was performed. Images were collected by Tannon5200 workstation. Image J software was used to analyze the optical density of the detected protein. The relative expression level of the detected protein was expressed by the gray ratio of the detected protein to GAPDH.

### Statistical Analysis

All values are expressed as the mean ± standard error. Student-s t test was performed with GraphPad Prism software (San Diego, California) to evaluate statistical significance and one-way analysis of variance (ANOVA) was used to analyze comparisons among multiple groups. But when there are two variables, we use two-way ANOVA. When P<0.05, it is considered to be statistically significant. The data are statistically evaluated by variance analysis, and then Prism is used for Tukey post-test of inter-group comparison.

## Results

### Biochemical Variables

We verified the success of DM modeling through glucose tolerance and insulin resistance index. The results showed that: compared with the Control group, the blood sugar of the STZ group and STZ+H_2_S group were significantly increased (P<0.05) (**Table 1**), suggesting diabetic glucose tolerance; at the same time, we also found that the fasting blood glucose, fasting insulin level and insulin resistance index of the STZ group and STZ+H_2_S group were significantly Elevated (P<0.05) (**Table 2**); the above results suggest that the type 2 diabetes model was successfully constructed.

**Table 1:** Expression of blood sugar in rats of each group.

Groups	0.5 h (mmol/L)	1 h (mmol/L)	2 h (mmol/L)
**Control**	5.57 ± 0.24	5.68 ± 0.37	4.27 ± 0.16
**STZ**	31.36 ± 0.62	31.09 ± 0.40	27.59 ± 1.34*
**STZ+H_2_S**	27.53 ± 2.56	30.7 ± 1.58	26.51 ± 3.40*

*p < 0.05 vs Control group. Values are expressed as mean ± SD.

**Table 2:** Expression of the fasting blood glucose, fasting insulin level and insulin resistance index(HOMA-IR) in rats of each group.

Groups	Fasting blood glucose	Fasting insulin level	HOMA-IR
	(mmol/L)	(uIU/ml,10^3^)	
**Control**	3.70 ± 0.32	25.00 ± 5.00	4.00 ± 0.80
**STZ**	23.15 ± 1.49*	362.00 ± 79.46*	363.03 ± 68.61*
**STZ+H_2_S**	22.89 ± 2.86*	350.00 ± 103.09*	341.82 ± 132.92*

*p < 0.05 vs Control group. Values are expressed as mean ± SD.

### Changes in the Content of Endogenous Hydrogen Sulfide (H_2_S) Synthase Cystathioether-Lyase (CSE)

**Figure 1:**
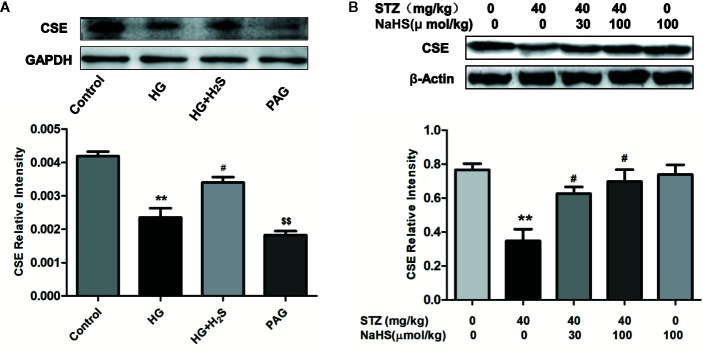
Exogenous hydrogen sulfide can up-regulate CSE expression levels. **(A)** Western blot analysis of CSE expression in H9c2 Cells; n = 3, **p < 0.01 vs control. ^#^p < 0.05 vs HG. ^$$^p < 0.01 vs HG+H_2_S. **(B)** Western blot analysis of CSE expression in myocardial tissue of diabetic rats; n = 3, **p < 0.01 vs control. ^#^p < 0.05 vs HG. HG, high glucose; H_2_S, hydrogen sulfide; PAG, dl-propargylglycine. STZ, streptozotocin.

The expression level of endogenous H_2_S synthetase CSE in high glucose induced H9c2 cardiomyocytes and myocardial tissue of diabetic rats was detected by Western blotting, The results showed that in cultured H9c2 cardiomyocytes, the expression level of CSE protein in HG group was significantly down-regulated compared with that in Control group (P<0.05), while H_2_S-treated could reverse the down-regulation of CSE expression level (P<0.05), and the expression of CSE protein in PAG group was also significantly down-regulated compared with that in Control group (P < 0.05). (**Figure 1A**) In vivo experiments, we also found that the expression level of CSE in the heart tissue of DM rats was significantly lower than that of the control group (P<0.05), while high and low concentrations of H_2_S intervention could up-regulate the expression level of CSE protein in the heart tissue of DM rats (P < 0.05) (**Figure 1B**), the above data show that high glucose stimulation can lead to down-regulation of endogenous H_2_S synthase CSE level in myocardial cells, while exogenous H_2_S can up-regulate CSE expression level.

### H_2_S Can Improve Cardiac Function in Diabetic Rats

The structure and function of left ventricle were observed by color Doppler echocardiography. The results showed that LVFS value decreased (P<0.05), LVEDD value and LVESD value increased (P<0.05) in STZ group compared with control group; However, in STZ+H_2_S group, LVFS value increased (P<0.05), LVEDD value and LVESD value decreased (P<0.05), suggesting that cardiac function improved. There was no significant difference in LVEDD, LVSED and LVFS between H_2_S group and Control group (P>0.05) (**Table 3**).

**Table 3:** Effects of H2S on Left ventricular end-diastolic dimension (LVEDD); left ventricular end-systolic diameter (LVESD); ejection fraction (EF); fractional shortening (FS) in rats.

Group	LVEDD (mm)	LVESD (mm)	EF (%)	FS (%)
**Control**	5.18 ± 0.40	2.65 ± 0.42	85.18 ± 4.04	49.03 ± 5.25
**STZ**	6.83 ± 0.37	4.80 ± 0.24*	63.57 ± 5.01*	30.03 ± 3.60*
**STZ+H_2_S**	5.84 ± 0.26^#^	3.73 ± 0.19^#^	73.07 ± 4.11^#^	35.93 ± 3.45^#^
**H_2_S**	5.30 ± 0.29	2.90 ± 0.36	83.08 ± 4.66	46.03 ± 4.41

*p < 0.05 vs Control group; ^#^p < 0.05 vs STZ group. Values are expressed as mean ± SD.

### High Glucose Can Induce Myocardial Cell Senescence and Aggravate Diabetic Myocardial Fibrosis, Which Can Be Antagonized by H_2_S

**Figure 2:**
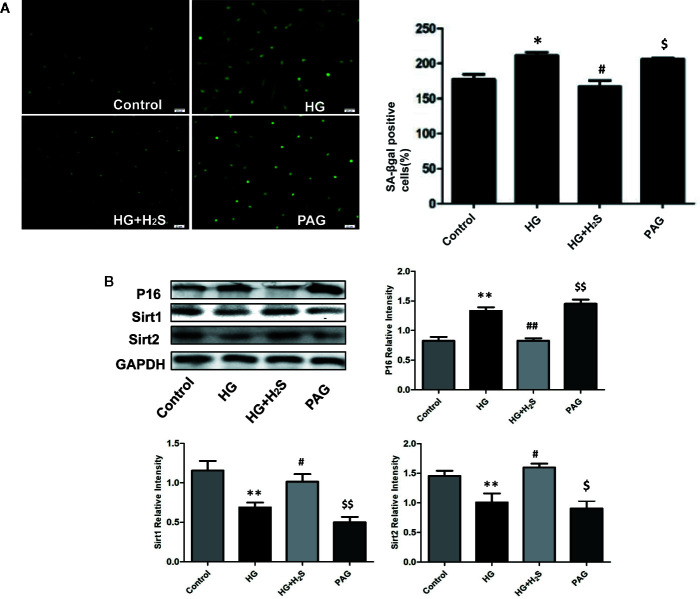
**(A)** Fluorescence microscope with green indicator analysis and quantitative results of senescent cells content in H9c2 cells treated with control; HG; HG+H_2_S; H_2_S; **(B)** Western blotting analysis the expression of P16; Sirt1; Sirt2 in H9c2 cells treated with Control; HG; HG+H_2_S; H_2_S; n = 3, *p < 0.05 vs control. **p < 0.01 vs control. ^#^p < 0.05 vs HG. ^##^p < 0.01 vs HG. ^$^p < 0.05 vs HG+H_2_S. ^$$^p < 0.01 vs HG+H2S. HG, High Glucose; H_2_S, hydrogen sulfide; PAG, dl-propargylglycine.

Some studies have found that myocardial cell aging participates in the mechanism of myocardial fibrosis, Therefore, this study observed whether high glucose stimulation can induce aging of H9c2 myocardial cells through *in vitro* experiments: The results of galactosidase staining showed that more H9c2 cardiomyocytes in HG group were senescent than those in control group, It is suggested that HG stimulation significantly intensifies the aging of H9c2 myocardial cells (**Figure 2A**) (P<0.05). However, the number of aged cardiomyocytes in HG+H_2_S group decreased significantly, while the number of aged cardiomyocytes in PAG group increased significantly (P<0.05). The expression levels of aging-related protein P16 were also observed by Western blotting method (P<0.05), and similar observation results were obtained, While The expression of senescence-inhibiting proteins Sirt1 and Sirt2 in HG was significantly down-regulated and hydrogen sulfide could antagonize the effect (**Figure 2B**). The above data show that HG stimulation can induce aging of H9c2 cardiomyocytes, while exogenous H_2_S can inhibit HG-induced cell aging.

**Figure 3:**
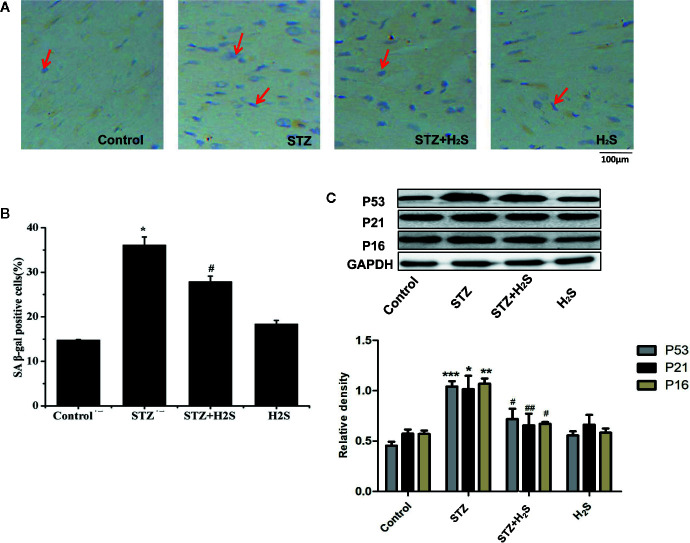
**(A)** Proportion of SA-β-gal positive cells in myocardial tissue in treated with Control; STZ; STZ+H_2_S; H_2_S (Red arrow represents SA-β-gal positive cells); **(B)** Quantitative results of SA-β-gal fluorescence intensity in myocardial tissue treated with Control; STZ; STZ+H_2_S; H_2_S; **(C)** Western blotting analysis the expression of P53, P21, P16 in myocardial tissue treated with Control; STZ; STZ +H_2_S; H_2_S; n = 3, *p < 0.05 vs control. **p < 0.01 vs control. ***p < 0.001 vs control. ^#^p < 0.05 vs STZ. ^##^p < 0.01 vs STZ. STZ, streptozotocin; H_2_S, hydrogen sulfide.

**Figure 4:**
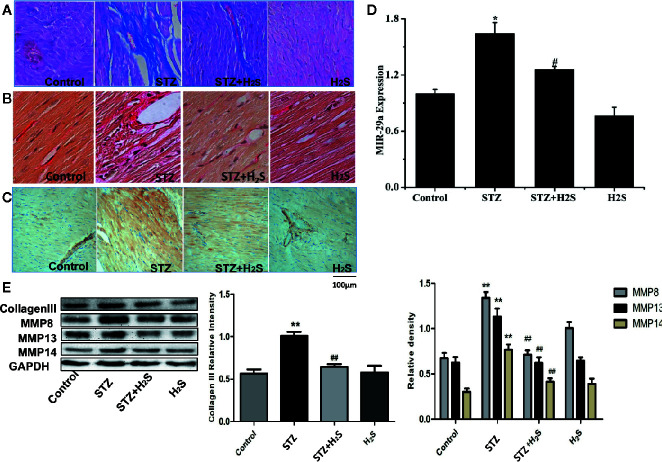
Upregulation of cardiac production of myocardial collagen fibers, type III collagen in STZ-injected rat *in vivo*, This effect can be antagonized by hydrogen sulfide **(A)** Representative images of masson with a myocardial collagen fibers in the heart tissues of STZ-injected rat models. Images were acquired at 10×40 magnification. **(B)** Representative images of VG with a myocardial collagen fibers in the heart tissues of STZ-injected rat models. Images were acquired at 10×40 magnification. **(C)** Representative images of immunocytochemical with myocardial collagen fibers in the heart tissues of STZ-injected rat models. Images were acquired at 10×40 magnification. **(D)** Comparison of expression of miR-29 in myocardial tissue treated with Control; STZ; STZ +H2S; H_2_S; **(E)** Western blotting analysis the expression of MMP8, MMP13, MMP14 in myocardial tissue treated with Control; STZ; STZ +H_2_S; H_2_S; n = 3, *p < 0.05 vs control. **p < 0.01 vs control. ^#^p < 0.05 vs STZ. ^##^p < 0.01 vs STZ. STZ, streptozotocin; H_2_S, hydrogen sulfide.

In vivo experiments, We saw that the β-galactosidase staining results of the STZ group rats compared with the control group showed that the myocardial cells of the STZ group were senescent (**Figures 3A, B**) (P<0.05), it was also seen that the expression levels of aging-related proteins P53, P21 and P16 in myocardial tissue of STZ group were significantly increased compared with the control group (**Figure 3C**) (P<0.05). At the same time, Masson staining also showed that myocardial collagen fiber deposition increased significantly (**Figure 4A**), while H_2_S intervention could reverse the above related changes of cell senescence, and myocardial tissue collagen fiber deposition also improved significantly compared with the previous. VG staining also confirmed similar changes of the above collagen deposition (**Figure 4B**), At the same time, immunohistochemistry also showed a significant increase in type III collagen observed by immunohistochemistry (**Figure 4C**); RT-qPCR analysis also showed that the relevant miRNA29 involved in fibrosis regulation in myocardial tissue of diabetic rats were significantly up-regulated (P<0.05) (**Figure 4D**), while the expression levels of the above miRNA29 were significantly down-regulated (P<0.05) after H_2_S intervention; The results of Western blotting also showed that the expression of type III collagen and MMP-8, MMP-13, MMP-14 were significantly up-regulated (P<0.05) (**Figure 4E**). H_2_S intervention could reverse the expression of the above-mentioned related proteins. In conclusion, the above evidence shows that myocardial cell aging is closely related to the occurrence and development of myocardial fibrosis in diabetic rats, while H_2_S intervention can improve cell aging and diabetic myocardial fibrosis.

### H_2_S Can Inhibit Myocardial Cell Senescence and Diabetic Myocardial Fibrosis by Up-Regulating Autophagy

**Figure 5:**
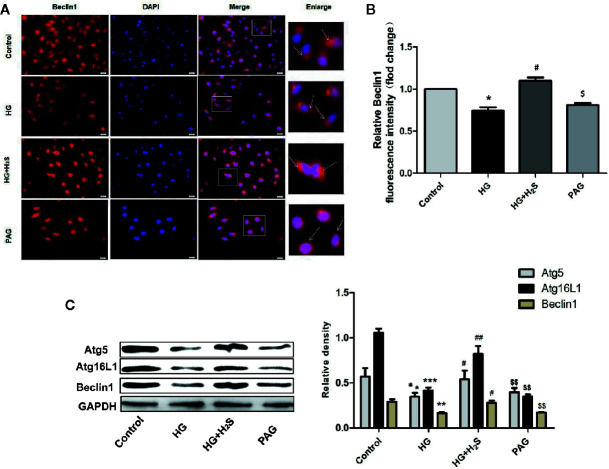
**(A)** Fluorescence microscope with Beclin1 Red Probe analysis and quantitative results of autophagy intensity in H9c2 cells treated with Control; HG; HG +H_2_S; H_2_S. **(B)** Quantitative results of BECLIN1 red fluorescence intensity in H9c2 treated with Control; HG; HG +H_2_S; H_2_S. Data were normalized to control. **(C)** Western blotting analysis the expression of Atg5, Atg12, Atg16L1 and Beclin1 in H9c2 cells treated with Control; HG; HG +H_2_S; H_2_S. n = 3; *p < 0.05 vs control, **p < 0.01 vs control, ***p < 0.001 vs control, ^#^p < 0.05 vs HG, ^##^p < 0.01vs HG, ^$^p < 0.05 vs HG+H_2_S; ^$$^p < 0.01 vs HG+H2S; HG, high glucose; H_2_S, hydrogen sulfide; PAG, dl-propargylglycine.

In this study, the changes of autophagy of H9c2 myocardial cells were observed by immunofluorescence method. As shown in **Figures 5A, B**, autophagy of H9c2 myocardial cells in HG group was significantly down-regulated, while autophagy was significantly enhanced after H_2_S-treated (P<0.05). On the contrary, after PAG inhibited endogenous H_2_S production, autophagy also showed a downward trend (P<0.05). At the same time, the changes of autophagy-related protein expression were observed by immunoblotting. It was found that the protein expression levels of Atg5, Atg16L1, and Beclin1 in H9c2 cardiomyocytes in HG group were down-regulated, while H_2_S-treated could reverse the changes of the above autophagy-related protein expression (P < 0.05). (**Figure 5C**)

**Figure 6:**
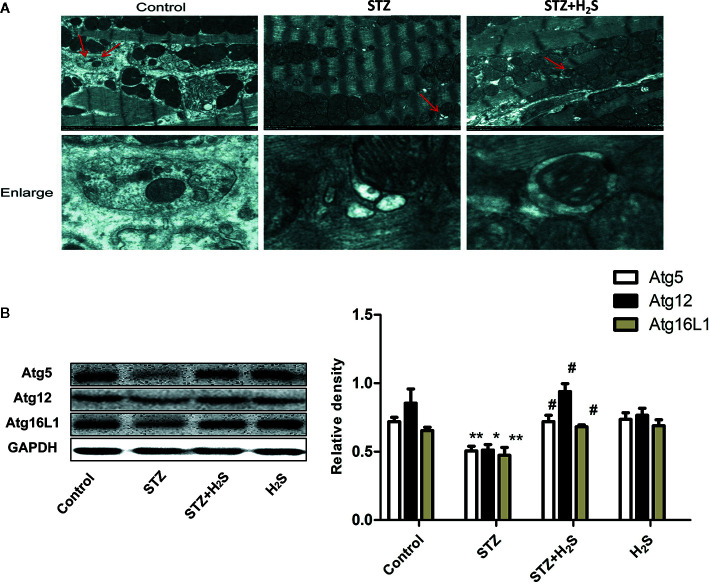
H2S upregulates STZ-induced diabetic myocardial fibrosis mitophagy. **(A)** Representative images of transmission electron microscopy analysis the effect of STZ‐induced diabetic myocardial fibrosis in STZ-evoked mitophagy in the selected area. The insets in the above panels are magnified and are presented in the lower panel. Transmission electron micrographs at bars of 0.2 µm(Red arrow represents microscopy). **(B)** Western blotting analysis the expression of Atg5,Atg12,Atg16L1 in myocardial tissue treated with Control; STZ; STZ +H2S; H2S; n = 3, *p < 0.05 vs control. **p < 0.01 vs control. ^#^p < 0.05 vs STZ. STZ, streptozotocin; H2S, hydrogen sulfide.

In this study, autophagy and ultrastructural changes of mitochondria in myocardial cells of rats in each group were also observed by transmission electron microscope. The results showed that the mitochondrial morphology of myocardial cells in the control group was normal, and a certain number of autophagy bodies could be observed (**Figure 6A**, above). However, myocardial cells in STZ group showed obvious mitochondrial morphological changes, such as cristae swelling and disorder, and the number of autophagy bodies in visual field decreased significantly. However, H_2_S-treated can improve mitochondrial morphological changes in myocardial cells of diabetic rats and increase the number of autophagy bodies (**Figure 6A**, bottom). The expression of autophagy-related proteins Atg5, Atg12, Atg16L1 in myocardium of rats in each group was also detected by Western blot protein detection method (P<0.05), and similar changes were also seen. All these results suggest that high glucose stimulation can induce down-regulation of autophagy level of myocardial cells, while H_2_S-treated can activate autophagy of cells. (**Figure 6B**).

### H_2_S Activates Autophagy Through SIRT6/AMPK to Improve Myocardial Cell Aging and Diabetic Myocardial Fibrosis

**Figure 7:**
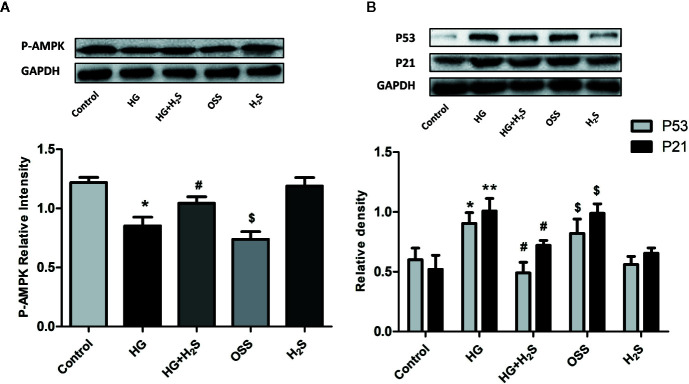
**(A)** Western blotting analysis the expression of P-AMPK in H9c2 cells treated with Control; HG; HG+H2S; OSS;H2S; n = 3, *p < 0.05 vs control. ^#^p < 0.05 vs HG. ^$^p < 0.05 vs HG+H2S; **(B)** Western blotting analysis the expression of P21; P53 in H9c2 cells treated with control; HG; HG+H_2_S; OSS; H_2_S; n = 3, *p < 0.05 vs control. **p < 0.01 vs control. ^#^p < 0.05 vs HG. ^$^p < 0.05 vs HG+H2S; HG, high glucose; H_2_S, hydrogen sulfide; OSS, Sirt6 inhibitor OSS_128167.

**Figure 8:**
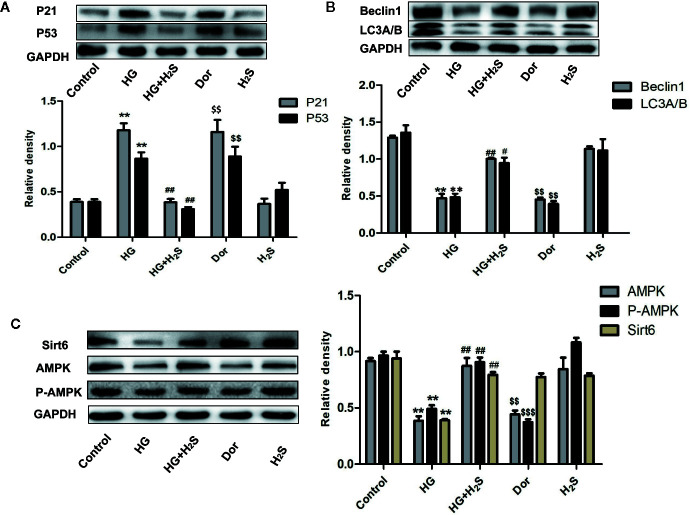
**(A)** Western blotting analysis the expression of P21; P53 in H9c2 cells treated with Control; HG; HG+H_2_S; Dor; H_2_S; n = 3, **p < 0.01 vs control. ^##^p < 0.01 vs HG. ^$$^p < 0.01 vs HG+H2S; **(B)** Western blotting analysis the expression of Beclin1; LC3A/B in H9C2 cells treated with Control; HG; HG+H_2_S; Dor;H_2_S; n = 3, **p < 0.05 vs control. ^#^p < 0.05 vs HG. ^##^p < 0.01 vs HG. ^$$^p < 0.01 vs HG+H2S; **(C)** Western blotting analysis the expression of Sirt6, AMPK, P-AMPK in H9C2 cells treated with Control; HG; HG+H_2_S; Dor;H2S; n = 3, **p < 0.01 vs control. ^##^p < 30.01 vs HG. ^$$^p < 0.01 vs HG+H2S; ^$$$^p < 0.01 vs HG+H2S; HG, high glucose; H_2_S, hydrogen sulfide; Dor, dorsomorphin dihydrochloride.

In vitro, it was found that HG could induce myocardial cell senescence, and the expression levels of Sirt6/AMPK signaling pathway proteins Sirt6 and AMPK protein were significantly down-regulated (P<0.05) while hydrogen sulfide intervention could up-regulate the expression level of Sirt6/AMPK. In order to further investigate whether Sirt6/AMPK signaling pathway is involved in the regulation mechanism of H_2_S improving myocardial cell senescence, we added Sirt6 inhibitor OSS_128167 (**Figure 7A**) or AMPK inhibitor (**Figure 8A**), Dorsomorphin dihydrochloride to H_2_S+HG group. The results were consistent with our expectation. Sirt6 inhibitor or AMPK inhibition reversed the autophagy activation and senescence inhibition of H_2_S. Autophagy-related proteins LC3A/B and Beclin1 were down-regulated in H_2_S+HG+Dor group (P<0.05) (**Figure 8B**), the same as H_2_S+HG+OSS group (P<0.05), (**Figure 8B**). While senescence-related proteins P53 and P21 were significantly up-regulated (P<0.05) (**Figures 7B** and **8C**). It is suggested that H_2_S can improve myocardial cell senescence by activating autophagy through SIRT6/AMPK.

**Figure 9:**
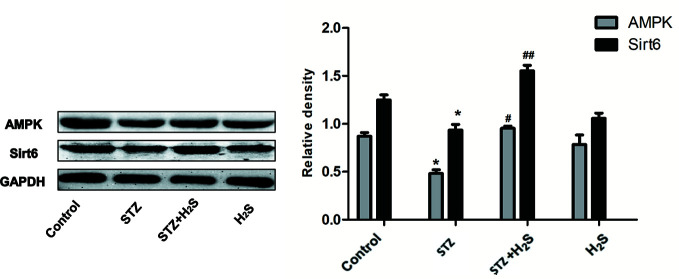
Western blotting analysis the expression of Sirt6; AMPK in STZ-induced diabetic myocardial fibrosis treated with Control; STZ; STZ +H_2_S; H_2_S; n = 3, *p < 0.05 vs control. ^#^p < 0.05 vs STZ. ^##^p < 0.01 vs STZ. STZ, streptozotocin; H2S,hydrogen sulfide.

Similarly, in STZ-induced diabetic rat model, Western blotting method also found that the expression of Sirt6/AMPK signaling pathway related proteins Sirt6, AMPK and other proteins in myocardial tissue of diabetic rats was significantly down-regulated (P<0.05) (**Figure 9**), and H_2_S-treated could up-regulate the expression of the above proteins. This is consistent with our results *in vitro*. The above results suggest that H_2_S can improve myocardial cell aging and diabetic myocardial fibrosis, and its mechanism may be to activate autophagy through SIRT6/AMPK pathway.

## Discussion

Diabetes mellitus is one of the common chronic diseases that seriously endanger human health, which can lead to structural and functional damage to multiple organs of the whole body including the heart. The adverse prognosis of diabetes mellitus is closely related to cardiovascular complications. DCM, as one of the major complications of diabetes mellitus, is an important origin leading to high incidence and mortality of diabetes-related cardiovascular diseases (Zamora and Villena, 2019). Myocardial fibrosis is a well-established cause and key factor for the increased myocardial stiffness and subsequent diastolic dysfunction in the diabetic patients (Hu et al., 2017; Parim et al., 2019).It has been found that aging participates in the mechanism of cardiovascular remodeling (Lakatta and Levy, 2003), including myocardial fibrosis and electrophysiological remodeling (Swynghedauw, 1999; Martin et al., 2019). HUA and others have found that ROS accumulation was achieved as myocardial cell aging, leading to myocardial remodeling (Hua et al., 2015). However, inhibition of myocardial cell aging or removal of aging myocardial cells can improve myocardial fibrosis and myocardial remodeling (Yan et al., 2019; Anderson et al., 2019). At the same time, inhibition of myocardial cell aging was probably found to be an important intervention link in diabetic myocardial fibrosis, but the related mechanism is not clear until now (Rawal et al., 2017). In this study, diabetic rat model established by intraperitoneal injection of STZ demonstrates distinct myocardial fibrosis and cell aging in diabetic rat myocardium. At the same time, it is showed that HG-induced H9c2 myocardial cells have significantly more premature cell aging *in vitro* experiments. Therefore, premature aging of cardiomyocytes presumably leads to an important mechanism for the development of diabetic myocardial fibrosis.

Autophagy is a metabolic pathway widely existing in eukaryotic cells that degrades its own components through lysosomal mechanism. By autophagy, cells can degrade damaged organelles and macromolecules, So as to prolong cell life, Autophagy dysfunction is one of the important characteristics of heart aging (Mizushima and Komatsu, 2011). However, the characteristics of terminal differentiation of myocardial cells make it difficult for them to remove damaged or excess organelles and macromolecules in cells through cell division like other cells. Therefore, autophagy plays an important role in maintaining health and homeostasis of myocardial cells, and autophagy disorder may lead to decrease of cell homeostasis (Shirakabe et al., 2016).On the contrary, enhancing autophagy activity may contribute to the survival of myocardial cells (Li et al., 2015). In this study, through STZ-induced diabetic rat model and HG-induced H9c2 myocardial cells. There is obvious premature cell aging in myocardial cells stimulated by high glucose, which is associated with the down-regulation of autophagy level. This shows that the down-regulation of autophagy level can further destroys the physiological homeostasis of myocardial cells under high glucose stress, resulting in a large accumulation of damaged and aging mitochondria, ultimately premature aging of cardiomyocytes is induced, leading to local myocardial inflammation and myocardial fibrosis through SASP.

Human silencing regulatory protein 6 (SIRT6) is a member of the sirtuins family SIRT1-SIRT7 in mammals. SIRT6 is located in the nucleus and is a protease with various catalytic activities, which has histone deacetylation, ADP ribosylation and lysine deacylase effects. SIRT6 plays an important role in DNA repair and maintenance of genome stability, Participating in various physiological regulation mechanisms such as cell proliferation, differentiation and aging (Gertler and Cohen, 2013), Recent studies have shown that SIRT6 can up-regulate AMP/ATP, then activate AMPK/Fox O3, Exerting antioxidant stress effect (Wang et al., 2016), Fan et al. observed that overexpression of Sirt6 can increase AMPK phosphorylation level, The apoptosis of podocyte induced by HG was reduced (Fan et al., 2019).At the same time, some studies have revealed that SIRT6 overexpression can inhibit senescence and apoptosis of nucleus pulposus cells by inducing autophagy (Chen et al., 2018). Some studies pointed out that AMPK activity in aged and aging hearts is significantly reduced compared with young hearts, AMPK is an important regulatory kinase upstream of myocardial m TOR, which can inhibit the kinase activity of mTOR and activate autophagy to protect myocardium (Ma et al., 2011). Similarly, Ning and other scholars have confirmed that increasing AMPK protein expression in renal tissue of aged male rats can promote autophagy and delay renal aging (Ning et al., 2013). The results of this study show that HG-induced stress senescence of H9c2 cardiomyocytes is obvious, autophagy level is also significantly down-regulated, SIRT6/AMPK signaling pathway is remarkably inhibited, and the expression levels of related proteins such as Sirt6, AMPK, P-AMPK are all down-regulated. Similarly, identical changes were also found in STZ-induced diabetic rat models. Therefore, the above results suggest that myocardial cell aging participates in the mechanism of diabetic myocardial fibrosis, which is associated with the down-regulation of SIRT6/AMPK/autophagy level of myocardial cells.

Hydrogen sulfide (H_2_S), as the third newly discovered endogenous gas messenger molecule in recent years, is widely distributed in various parts of the body. CSE is the key enzyme of endogenous H_2_S source in cardiovascular system (Zheng et al., 2018), CBS and 3MST also participate in the production of endogenous H_2_S in peripheral vascular tissue (Prabhudesai et al., 2018). Our research group has confirmed that H_2_S could antagonize myocardial fibrosis by regulating multiple intracellular signaling pathways (Liu et al., 2018a; Liu et al., 2018b), however the internal regulatory mechanism of H_2_S is very complex, which is still not clear until now. Therefore, it is of great significance to further explore the myocardial protection mechanism of H_2_S. This study observed that exogenous H_2_S can activate endogenous H_2_S-producing enzyme CSE and improve STZ-induced myocardial fibrosis and MMPs/TIMP1 imbalance in diabetic rats. In vitro and *in vivo* experiments, we found that H_2_S can significantly up-regulate autophagy level, inhibit myocardial cell aging and premature heart aging after HG-induced cardiomyocyte aging, and its mechanism may be related to activation of SIRT6/AMPK signaling pathway. In order to further explore whether the mechanism of H_2_S inhibiting myocardial cell senescence is related to the activation of SIRT6/AMPK signaling pathway. We added AMPK inhibitor Dorsomorphin dihydrochloride (Dor group) to H_2_S-treated group *in vitro*, and the results were consistent with our expectations. The autophagy level of myocardial cells in Dor group was down-regulated and the expression of aging-related proteins was significantly increased, suggesting that H_2_S inhibits premature aging of H9c2 myocardial cells by activating autophagy of cells through SIRT6/AMPK.

## Conclusion

In summary, exogenous H_2_S was found to activate autophagy through SIRT6/AMPK signaling pathway, the senescence of myocardial cell was inhibited and diabetic myocardial fibrosis was improved. The molecular mechanism of HG-induced aging of H9c2 cardiomyocytes and myocardial fibrosis in diabetic rats was further discussed. It is revealed that activation of autophagy and inhibition of cardiomyocyte senescence through SIRT6/AMPK pathway probably is a noval mechanism for H_2_S to improve diabetic myocardial fibrosis, and SIRT6/AMPK/autophagy is likely to be a new prevention and treatment of cardiomyocyte aging and diabetic myocardial fibrosis target, endogenous H_2_S is likely to be an important part of antagonizing premature aging of myocardial cells and myocardial remodeling, more internal regulatory mechanisms need to be further studied and discussed.

## Data Availability Statement

The original contributions presented in the study are included in the article/supplementary material; further inquiries can be directed to the corresponding authors.

## Ethics Statement

The animal study was reviewed and approved by the University Committee on the Use and Care of Animals of South China University.

## Author Contributions

YL, ML, XS, XZ, CC, and JYa were involved in study design and writing the manuscript. JYi, DL, and SW contributed to the experiment and data analysis. All authors contributed to the article and approved the submitted version.

## Funding

This study was supported by grant from the National Natural Science Foundation of China (Grant Nos. 81870230); Hunan Natural Science Foundation (2019JJ80033) and Health Scientific Research Fund Project of Hunan Provincial Health Commission (20190225).

## Conflict of Interest

The authors declare that the research was conducted in the absence of any commercial or financial relationships that could be construed as a potential conflict of interest.

## References

[B1] Anderson R. Lagnado A. Maggiorani D. Walaszczyk A. Dookun E. Chapman J. (2019). Length-independent telomere damage drives post-mitotic cardiomyocyte senescence. EMBO J. 38 (5), e100492. 10.15252/embj.2018100492 30737259 PMC6396144

[B2] Annoni G. Luvara G. Arosio B. Gagliano N. Fiordaliso F. Santambrogio D. (1998). Age-dependent expression of fibrosis-related genes and collagen deposition in the rat myocardium. Mech. Ageing Dev. 101 (1-2), 57–72. 10.1016/s0047-6374(97)00165-6 9593313

[B3] Arumugam T. V. Kennedy B. K. (2018). H2S to Mitigate Vascular Aging: A SIRT1 Connection. Cell 173 (1), 8–10. 10.1016/j.cell.2018.03.011 29571000

[B4] Chao P. C. Li Y. Chang C. H. Shieh J. P. Cheng J. T. Cheng K. C. (2018). Investigation of insulin resistance in the popularly used four rat models of type-2 diabetes. BioMed. Pharmacother. 101, 155–161. 10.1016/j.biopha.2018.02.084 29486333

[B5] Chen J. Xie J. J. Jin M. Y. Gu Y. T. Wu C. C. Guo W. J. (2018). Sirt6 overexpression suppresses senescence and apoptosis of nucleus pulposus cells by inducing autophagy in a model of intervertebral disc degeneration. Cell Death Dis. 9 (2), 56. 10.1038/s41419-017-0085-5 29352194 PMC5833741

[B6] Doura T. Kamiya M. Obata F. Yamaguchi Y. Hiyama T. Y. Matsuda T. (2016). Detection of LacZ-Positive Cells in Living Tissue with Single-Cell Resolution. Angew Chem. Int. Ed. Engl. 55 (33), 9620–9624. 10.1002/anie.201603328 27400827

[B7] Elhanati S. Kanfi Y. Varvak A. Roichman A. Carmel-Gross I. Barth S. (2013). Multiple regulatory layers of SREBP1/2 by SIRT6. Cell Rep. 4 (5), 905–912. 10.1016/j.celrep.2013.08.006 24012758

[B8] Falcao-Pires I. Leite-Moreira A. F. (2012). Diabetic cardiomyopathy: understanding the molecular and cellular basis to progress in diagnosis and treatment. Heart Fail Rev. 17 (3), 325–344. 10.1007/s10741-011-9257-z 21626163

[B9] Fan Y. Yang Q. Yang Y. Gao Z. Ma Y. Zhang L. (2019). Sirt6 Suppresses High Glucose-Induced Mitochondrial Dysfunction and Apoptosis in Podocytes through AMPK Activation. Int. J. Biol. Sci. 15 (3), 701–713. 10.7150/ijbs.29323 30745856 PMC6367578

[B10] Gertler A. A. Cohen H. Y. (2013). SIRT6, a protein with many faces. Biogerontology 14 (6), 629–639. 10.1007/s10522-013-9478-8 24213807

[B11] Glance L. G. Dick A. W. Glantz J. C. Wissler R. N. Qian F. Marroquin B. M. (2014). Rates of major obstetrical complications vary almost fivefold among US hospitals. Health Aff. (Millwood) 33 (8), 1330–1336. 10.1377/hlthaff.2013.1359 25092833

[B12] Guo Y. Zhuang X. Huang Z. Zou J. Yang D. Hu X. (2018). Klotho protects the heart from hyperglycemia-induced injury by inactivating ROS and NF-kappaB-mediated inflammation both in vitro and in vivo. Biochim. Biophys. Acta Mol. Basis Dis. 1864 (1), 238–251. 10.1016/j.bbadis.2017.09.029 28982613

[B13] Hu X. Bai T. Xu Z. Liu Q. Zheng Y. Cai L. (2017). Pathophysiological Fundamentals of Diabetic Cardiomyopathy. Compr. Physiol. 7 (2), 693–711. 10.1002/cphy.c160021 28333387

[B14] Hua Y. Robinson T. J. Cao Y. Shi G. P. Ren J. Nair S. (2015). Cathepsin K knockout alleviates aging-induced cardiac dysfunction. Aging Cell 14 (3), 345–351. 10.1111/acel.12276 25692548 PMC4406663

[B15] Kanwal A. Pillai V. B. Samant S. Gupta M. Gupta M. P. (2019). The nuclear and mitochondrial sirtuins, Sirt6 and Sirt3, regulate each other’s activity and protect the heart from developing obesity-mediated diabetic cardiomyopathy. FASEB J. 33 (10), 10872–10888. 10.1096/fj.201900767R 31318577 PMC6766651

[B16] Kim J. H. Lee J. M. Kim J. H. Kim K. R. (2018). Fluvastatin activates sirtuin 6 to regulate sterol regulatory element-binding proteins and AMP-activated protein kinase in HepG2 cells. Biochem. Biophys. Res. Commun. 503 (3), 1415–1421. 10.1016/j.bbrc.2018.07.057 30078674

[B17] Koopman R. J. Mainous A. G. ,. 3. Diaz V. A. Geesey M. E. (2005). Changes in age at diagnosis of type 2 diabetes mellitus in the United States, 1988 to 2000. Ann. Fam Med. 3 (1), 60–63. 10.1370/afm.214 15671192 PMC1466782

[B18] Kwak H. B. Lee Y. Kim J. H. Van Remmen H. Richardson A. G. Lawler J. M. (2015). MnSOD overexpression reduces fibrosis and pro-apoptotic signaling in the aging mouse heart. J. Gerontol. A Biol. Sci. Med. Sci. 70 (5), 533–544. 10.1093/gerona/glu090 25016531 PMC4462657

[B19] Lakatta E. G. Levy D. (2003). Arterial and cardiac aging: major shareholders in cardiovascular disease enterprises: Part I: aging arteries: a “set up” for vascular disease. Circulation 107 (1), 139–146. 10.1161/01.cir.0000048892.83521.58 12515756

[B20] Li X. Zeng Z. Li Q. Xu Q. Xie J. Hao H. (2015). Inhibition of microRNA-497 ameliorates anoxia/reoxygenation injury in cardiomyocytes by suppressing cell apoptosis and enhancing autophagy. Oncotarget 6 (22), 18829–18844. 10.18632/oncotarget.4774 26299920 PMC4643066

[B21] Liu M. Li Z. Liang B. Li L. Liu S. Tan W. (2018a). Hydrogen sulfide ameliorates rat myocardial fibrosis induced by thyroxine through PI3K/AKT signaling pathway. Endocr. J. 65 (7), 769–781. 10.1507/endocrj.EJ17-0445 29743447

[B22] Liu M. Li Y. Liang B. Li Z. Jiang Z. Chu C. (2018b). Hydrogen sulfide attenuates myocardial fibrosis in diabetic rats through the JAK/STAT signaling pathway. Int. J. Mol. Med. 41 (4), 1867–1876. 10.3892/ijmm.2018.3419 29393353 PMC5810211

[B23] Ma H. Guo R. Yu L. Zhang Y. Ren J. (2011). Aldehyde dehydrogenase 2 (ALDH2) rescues myocardial ischaemia/reperfusion injury: role of autophagy paradox and toxic aldehyde. Eur. Heart J. 32 (8), 1025–1038. 10.1093/eurheartj/ehq253 20705694 PMC3076664

[B24] Martin B. Gabris B. Barakat A. F. Henry B. L. Giannini M. Reddy R. P. (2019). Relaxin reverses maladaptive remodeling of the aged heart through Wnt-signaling. Sci. Rep. 9 (1), 18545. 10.1038/s41598-019-53867-y 31811156 PMC6897890

[B25] Mizushima N. Komatsu M. (2011). Autophagy: renovation of cells and tissues. Cell 147 (4), 728–741. 10.1016/j.cell.2011.10.026 22078875

[B26] Ning Y. C. Cai G. Y. Zhuo L. Gao J. J. Dong D. Cui S. (2013). Short-term calorie restriction protects against renal senescence of aged rats by increasing autophagic activity and reducing oxidative damage. Mech. Ageing Dev. 134 (11-12), 570–579. 10.1016/j.mad.2013.11.006 24291536

[B27] Parim B. Sathibabu Uddandrao V. V. Saravanan G. (2019). Diabetic cardiomyopathy: molecular mechanisms, detrimental effects of conventional treatment, and beneficial effects of natural therapy. Heart Fail Rev. 24 (2), 279–299. 10.1007/s10741-018-9749-1 30349977

[B28] Prabhudesai S. Koceja C. Dey A. Eisa-Beygi S. Leigh N. R. Bhattacharya R. (2018). Corrigendum: Cystathionine beta-Synthase Is Necessary for Axis Development in vivo. Front. Cell Dev. Biol. 6:121:121. 10.3389/fcell.2018.00121 30310813 PMC6171515

[B29] Rawal S. Munasinghe P. E. Nagesh P. T. Lew J. K. S. Jones G. T. Williams M. J. A. (2017). Down-regulation of miR-15a/b accelerates fibrotic remodelling in the Type 2 diabetic human and mouse heart. Clin. Sci. (Lond) 131 (9), 847–863. 10.1042/CS20160916 28289072

[B30] Rota M. LeCapitaine N. Hosoda T. Boni A. De Angelis A. Padin-Iruegas M. E. (2006). Diabetes promotes cardiac stem cell aging and heart failure, which are prevented by deletion of the p66shc gene. Circ. Res. 99 (1), 42–52. 10.1161/01.RES.0000231289.63468.08 16763167

[B31] Shirakabe A. Ikeda Y. Sciarretta S. Zablocki D. K. Sadoshima J. (2016). Aging and Autophagy in the Heart. Circ. Res. 118 (10), 1563–1576. 10.1161/CIRCRESAHA.116.307474 27174950 PMC4869999

[B32] Swynghedauw B. (1999). Molecular mechanisms of myocardial remodeling. Physiol. Rev. 79 (1), 215–262. 10.1152/physrev.1999.79.1.215 9922372

[B33] Wang X. X. Wang X. L. Tong M. M. Gan L. Chen H. Wu S. S. (2016). SIRT6 protects cardiomyocytes against ischemia/reperfusion injury by augmenting FoxO3alpha-dependent antioxidant defense mechanisms. Basic Res. Cardiol. 111 (2), 13. 10.1007/s00395-016-0531-z 26786260

[B34] Wang Y. Li N. Jiang W. Deng W. Ye R. Xu C. (2018). Mutant LKB1 Confers Enhanced Radiosensitization in Combination with Trametinib in KRAS-Mutant Non-Small Cell Lung Cancer. Clin. Cancer Res. 24 (22), 5744–5756. 10.1158/1078-0432.CCR-18-1489 30068711

[B35] Wang Y. Zhang R. Shen H. Kong J. Lv X. (2019). Pioglitazone protects blood vessels through inhibition of the apelin signaling pathway by promoting KLF4 expression in rat models of T2DM. Biosci. Rep. 39 (12), BSR20190317. 10.1042/BSR20190317 PMC6928522 31829402

[B36] Xu W. Chen J. Lin J. Liu D. Mo L. Pan W. (2015). Exogenous H2S protects H9c2 cardiac cells against high glucose-induced injury and inflammation by inhibiting the activation of the NF-kappaB and IL-1beta pathways. Int. J. Mol. Med. 35 (1), 177–186. 10.3892/ijmm.2014.2007 25412187

[B37] Yan Y. Miao D. Yang Z. Zhang D. (2019). Loss of p27(kip1) suppresses the myocardial senescence caused by estrogen deficiency. J. Cell Biochem. 120 (8), 13994–14003. 10.1002/jcb.28674 30957908

[B38] Zamora M. Villena J. A. (2019). Contribution of Impaired Insulin Signaling to the Pathogenesis of Diabetic Cardiomyopathy. Int. J. Mol. Sci. 20 (11), 2833. 10.3390/ijms20112833 PMC6600234 31212580

[B39] Zheng S. F. Bao R. K. Zhang Q. J. Wang S. C. Lin H. J. (2018). Endogenous Hydrogen Sulfide Promotes Apoptosis via Mitochondrial Pathways in the Livers of Broilers with Selenium Deficiency Exudative Diathesis Disease. Biol. Trace Elem. Res. 186 (1), 249–257. 10.1007/s12011-018-1292-3 29524194

[B40] Zheng X. Peng M. Li Y. Wang X. Lu W. Wang X. (2019). Cathelicidin-related antimicrobial peptide protects against cardiac fibrosis in diabetic mice heart by regulating endothelial-mesenchymal transition. Int. J. Biol. Sci. 15 (11), 2393–2407. 10.7150/ijbs.35736 31595157 PMC6775320

[B41] Zhuo J. Zeng Q. Cai D. Zeng X. Chen Y. Gan H. (2018). Evaluation of type 2 diabetic mellitus animal models via interactions between insulin and mitogenactivated protein kinase signaling pathways induced by a high fat and sugar diet and streptozotocin. Mol. Med. Rep. 17 (4), 5132–5142. 10.3892/mmr.2018.8504 29393432 PMC5865978

